# Considering cross-cultural differences in sleep duration between Japanese and Canadian university students

**DOI:** 10.1371/journal.pone.0250671

**Published:** 2021-04-26

**Authors:** Benjamin Y. Cheung, Kosuke Takemura, Christine Ou, Anne Gale, Steven J. Heine

**Affiliations:** 1 University of British Columbia, Vancouver, Canada; 2 Shiga University, Shiga, Japan; 3 Kyoto University, Kyoto, Japan; Birkbeck University of London, UNITED KINGDOM

## Abstract

Sleep is a fundamental biological process that all humans exhibit, and there is much evidence that people suffer adverse health outcomes from insufficient sleep. Despite this evidence, much research demonstrates significant heterogeneity in the amounts that people sleep across cultures. This suggests that despite serving fundamental biological functions, sleep is also subject to cultural influence. Using self-report and actigraphy data we examined sleep among European Canadian, Asian Canadian, and Japanese university students. Significant cultural differences emerged in terms of various parameters of sleep (e.g. sleep time), and beliefs about sleep (e.g. perceived relation between sleep and health). Despite sleeping significantly less than European Canadians, Japanese participants slept less efficiently, yet reported being less tired and having better health. Moreover, relative to European Canadians, Japanese participants perceived a weaker relation between sleep and physical health, and had a significantly shorter ideal amount of sleep. Asian Canadians’ sleep behaviors and attitudes were largely similar to European Canadians suggesting that people acculturate to local cultural sleep norms.

## Considering cross-cultural differences in sleep duration

Sleep is a mental and physiological state characterized by particular patterns of biological activity such as changes in consciousness and environmental awareness, cycling of neurological activity, and inhibition of muscular movement [1]. Extensive research suggests that sleep is virtually a biological universal. Aside from humans, researchers have documented various forms of sleep across the animal kingdom, including nematodes such as *Caenorhabditis elegans* [2], insects such as *Drosophila melanogaster* [3], and primates such as *Macaca mulatta* [4]. Such widespread prevalence of sleep-like behaviors across the animal kingdom indicates that sleep has ancient evolutionary origins, and serves important biological functions, such as caloric conservation, cellular recovery, and promoting synaptic consolidation and plasticity [5–7]. Given the critical functions that have been attributed to sleep, it might seem reasonable to expect that people would sleep in highly similar ways across cultures. However, as we describe below, in many ways they do not. It remains a curious and largely unanswered question of how to make sense of the cultural variability in sleep practices.

### Cultural variability in sleep practices

Much research has revealed that sleep varies considerably across different cultural populations of humans [8]. Anthropological and epidemiological data suggest that cultures differ in many parameters of sleep such as sleep environments, sleep arrangements, and the segmentation of our sleep [9–13]. Underpinning such variability is the fact that human sleep is remarkably social in nature, and that sleep is not necessarily a behavior with a set of universally-applicable prescriptions and norms [14]. While a common norm among people living in Western industrialized societies is to sleep alone in a quiet, dark, climate-controlled space with soft bedding, this is a relatively recent development that stands in contrast to the sleep environments of many other non-Western, non-industrialized populations. Overall, ethnographic research has highlighted how much diversity there is in sleep practices around the world. For example, many foragers do not use any coverings and have minimal bedding (as this might harbor parasites), their sleep is often around a noisy fire which is tended throughout the night, in a social environment, with appendages entwined together, and they have fluid bedtimes with much nighttime activity [13]. This highly variable set of cultural sleep practices highlights how flexibly human sleep requirements can be satisfied.

One key way that sleep practices vary around the world is with regard for caretakers to cosleep with their children [15]. While this practice is relatively rare among European-descent Americans, and is in fact a rather controversial practice there [16, 17], it is quite common throughout much of the non-Western world. Indeed, one earlier review found that American parents were the only ones out of a survey of 100 societies who created a separate room for their babies to sleep in [18]. Cosleeping tends to be associated with reduced sleep duration [e.g., 19], and starkly different early formative experiences and expectations of young children’s sleep around the globe [17].

A second example of cultural variability is that, contrary to the current practice of a monophasic sleep period during the night found among people from industrialized countries through much of the world, biphasic sleep, including an afternoon siesta, is common in many small-scale societies [20, 21] and countries in the Mediterranean and South America [22]. However, these siesta cultures are more recently on the decline [23], despite the apparent decreased risk for coronary mortality among those who take a siesta [24]. Moreover, it appears that in the past much of human’s nighttime sleep patterns were likely divided into two long sessions that were separated by a period of wakefulness during which people were quite active [25, 26; but see 27]. This can be seen in historical records from Europe, with sleep patterns shifting to the modern single uninterrupted period during the late 17th century in urban, upper class Europe, potentially due to industrialization and the advent of electric lighting [28]. Further corroboration for this can be seen in experimental research which finds that Americans resort to an interrupted sleep pattern when exposed to abbreviated photoperiods [29]. These data highlight the importance of the environment, both social and physical, in influencing our sleep.

Moreover, there is substantial cross-cultural variability in the duration of people’s sleep, and these differences have been identified at various points across the lifespan. In particular, people from a variety of East Asian cultures, especially from Japan, sleep considerably less than those from various non-Western cultures, and these differences have been found using a wide variety of methods. For example, a survey from 48 countries found the least sleep to exist in Japan, at 5 hours and 59 minutes, which was an hour and a half less than that obtained in New Zealand, at 7 hours and 30 minutes [30]. A cross-sectional survey was given to adults in 10 countries which revealed that Japanese slept the least, at 6 hours and 53 minutes, which was about an hour and a half less than that observed in Portugal, at 8 hours and 24 minutes [31]. A survey of sleep habits in 30 countries found that the least sleep was had by Koreans and Japanese at 7 hours and 49 minutes and 7 hours and 50 minutes respectively, which was about an hour less than that obtained by the French at 8 hours and 50 minutes [32]. Data from 45 countries regarding time use revealed that the least sleep was had by Japanese at 7 hours and 16 minutes which was a full 3 hours less than that had by Argentinians, at 10 hours and 16 minutes [33]. A smartphone app was used to estimate sleep habits in 20 different countries, revealing that the least sleep in the world was held by Singaporeans and Japanese at 7 hours and 24 minutes and 7 hours and 31 minutes respectively, which was about 40 minutes less that that had by the Netherlands, at 8 hours and 5 minutes [34]. A comparison of sleep times among university students in 24 countries found the least sleep to be from Japanese female students at 6 hours and 5 minutes, which was almost 2 hours shorter than of Bulgarian female students at 8 hours [35]. A meta-analysis of adolescent sleep data across 23 countries from the past 30 years showed that total sleep time in Asian countries was 40–60 minutes shorter than in North America, and 60–120 minutes shorter than in Europe and Australia [36]. Another study examining the sleep of infants and toddlers from 17 predominantly Asian and Western countries found that children from Asian countries slept significantly less than children from Western countries, with the least sleep being among Japanese infants and toddlers at 11 hours and 37 minutes, which was about one hour and 40 minutes less than that of New Zealand (13 hours and 19 minutes; [37]). In addition, numerous studies have documented that various samples of different Asian cultural backgrounds sleep less than those of Western cultural backgrounds (e.g., [38–40]). Moreover, the amount that Japanese sleep has been decreasing over the past few decades with a systematic review identifying a decrease of 24 minutes of sleep a night over a 40 year time span [41].

These cultural differences are rather striking in magnitude–most of the above surveys found that people from some East Asian countries slept about an hour less than those in some other Western nations. It is possible that some of these differences might be due to methodological artifacts, such as cultural differences in response styles, given that the total estimated sleeping time varies so much from study to study (for example, estimates of Japanese sleeping time were almost two hours longer in calculations by [32], than in those by [30]). However, the finding that East Asians, and particularly, Japanese, so consistently anchor the low end of sleep duration in the world across the various methods used to calculate sleep in these studies is difficult to explain in terms of methodological factors. To the extent these measurements of cultural differences in sleep duration are valid, they raise the question of why they exist. [37] pointed out that these kinds of cultural differences “raise more questions than answers” and offered the speculative question of “whether there are biological differences contributing to these results or if these differences are solely culturally-based” (p. 277). Thus far, the field has been relatively mute on this question.

### Costs of insufficient sleep

What makes the evidence for cultural variability in sleep so striking though, is that so much of the research on sleep has focused on the pronounced cognitive and health costs that are associated with shorter sleep durations. A recurring set of findings in research on sleep is that people who do not regularly get a full night’s sleep (usually described as 7–8 hours) are at risk for a number of serious problems (for a review see [7]). For example, when people are deprived of sleep they may suffer from a large array of cognitive impairments, including diminished alertness, vigilance, working memory capacity, and learning [42, 43]. There is also much evidence that a lack of sleep is directly associated with a variety of health problems, such as an increased risk for Type 2 diabetes (e.g., [44]), obesity [45], increased inflammation (e.g., [46]), poorer cardiovascular outcomes (e.g., [47]), and an overall shorter life span [45, 48]. It is noteworthy that while most of the above research was conducted in Western countries, several of these harmful effects of reduced sleep have also been found within Japanese samples (e.g., [49–54]).

The above data lead to two contrasting sets of findings within the sleep literature. On the one hand we see that people from some cultures, in particular, Japanese and other East Asian countries, appear to consistently sleep substantially less than those from many Western cultures. On the other hand, research finds that when people sleep less than the recommended amount of 7–8 hours per night they can suffer serious cognitive impairments and health consequences. Given these findings, one might expect that people from cultures with less average sleep would tend to suffer more than Westerners from the various ills associated with restricted sleep. However, in comparison with people in the US, Japanese have far lower rates of obesity (3.7% vs. 38.2%; [55]), Type 2 diabetes (5.7% vs. 10.8%; [56]), deaths due to ischaemic heart disease (Standardized mortality rates per 100,000 of 6.5 vs. 23.1; [57]); and they have the greatest life expectancy of any major industrialized country on the planet (85.3 years, compared with 80.0 years for the US; [58]). While it is clearly possible that there may well be other cultural protective factors (e.g., dietary differences; [59, 60]) that might be concealing the negative effects of reduced sleep among Japanese, it is not immediately evident that cultures with lower average rates of sleep are necessarily suffering more from those same health consequences.

### Cultural variation in attitudes towards sleep

Perhaps we can better understand why cultures vary as much as they do in their sleep practices by considering people’s attitudes towards sleep. It seems reasonable to expect that a key factor that influences how much people sleep is their beliefs about the benefits of sleep and what a normative amount of sleep should be. For example, there is some evidence that sleep duration is associated with the degree to which people view sleep as important [61]. Similarly, perceived social norms and social pressures about bedtimes also predict sleep duration [34]. The most prominent social norm in many Westernized cultures is to sleep for 8 hours straight because of a shared understanding that sufficient sleep is important for better health [62, 63]. A discourse about this norm is ubiquitous in Westernized cultures, with public figures such as Ariana Huffington and Jeff Bezos expounding the importance of sleeping for at least 8 hours [64, 65].

In contrast to this perspective, Japanese culture maintains quite different attitudes towards sleep. There is a commonly held view in Japanese culture that sleep should be sacrificed in order to lead a successful life, and such sacrifice leads one to become morally fortified [66]. For example, the anthropologist, Ruth Benedict, noted in her classic 1946 ethnography that Japanese viewed sleep as something that was more akin to a luxury than a necessity, highlighting that: “One is to consider sleep apart from questions of recuperation, rest, and recreation” ([67], p. 180). Similarly, a common expression among Japanese students preparing for their notoriously difficult university entrance exams is that one will “pass with four, fail with five (*yontou*, *goraku*),” which refers to a belief that sleeping more than 4 hours a night will leave insufficient time for studying [66]. Indeed, there appears to be far less concern about the consequences of too little sleep among Japanese compared with Americans (e.g., [68]). Such attitudes towards sleep can be traced back to early Chinese literature. For example, [69] noted that Confucian teachings emphasized that reducing sleep was viewed as a basic requisite for functioning efficiently. Likewise, the Buddhist text *Daimin Sanzou Hosuu*, classified the urge to sleep as something which was in need of being controlled [70]. In sum, Japanese (and some other East Asian cultures) appear to view sleep as something that should be restricted to allow for optimal performance.

Accompanying this apparently normative belief among Japanese that sleep can be sacrificed, is a compensatory tolerance for public napping. It is acceptable, and often expected, for people to doze off in public places–a behaviour known as *inemuri*, which translates literally as “to be present and asleep”; [66], whereas napping would seem to be done more in private in the West. For example, Japanese journalists once documented that 40 members of parliament were asleep during sessions, and quite remarkably, when asked about it, several seemed to take it for granted [71]. *Inemuri* is generally not frowned upon because it is widely seen to serve as proof that one has been working hard enough to the point of exhaustion (Steger, 2003).

### The present research

While it is apparent that some aspects of sleep vary across cultures, there have been few targeted investigations to make sense of this variability. For example, we know little about the degree to which people from different cultures compare in their perceptions about the relation between sleep and health, nor in what they would view as an ideal sleep duration. To date, some data exist surrounding self-reported sleep and cultural beliefs about sleep among adults across various cultural groups (e.g., [72–74]) and many studies investigate physiological measures of sleep within a single cultural environment [75, 76]. In contrast, very few studies combine both methodologies. That is, little research has examined sleep in cross-cultural contexts using physiological measures, in addition to examining cultural beliefs about sleep using quantitative tools.

This paper seeks to examine the extent of cultural differences among Japanese and Canadian university students in various common parameters of sleep including sleep duration and sleep efficiency, as well as to quantify cultural differences in perceptions of sleep, using a combination of physiological and self-report data. We focus on a comparison of Canada and Japan as past cross-national research has found that Japan typically has the shortest sleep duration in the world (e.g., [37, 32, 34]). In contrast, the sleep duration in Canada typically falls above the global mean, and is similar to that found in the most studied population, the US, which provides the basis for the Western viewpoint. We chose to focus on university students, as university life is recognized as a time in Japan with relatively fewer responsibilities, in comparison with the very busy time as a child spent preparing towards university entrance exams, and in the hectic work life as an adult [77, 78]; hence, we would expect that our Japanese participants would have relatively fewer obligations cutting into their sleep time than if we had surveyed Japanese samples from many other points in their life course. The study relied on actigraphy data as physiological measures of sleep duration and percentage of sleep time sleeping, while also using self-report measures to quantify various perceptions and cultural norms about sleep. Based on the different cultural norms regarding sleep between Canada and Japan and other East Asian countries, the participants consisted of European-Canadian, Asian-Canadian, and Japanese university students, representing various levels of connections with Westernized norms associated with sleep. By targeting East Asian samples, we can also explore the question of whether any biological factors associated with ethnic differences are likely involved in any population differences in sleep. The study was approved by the University of British Columbia Behavioral Research Ethics Board (H10-02590).

## Methods

### Participants

We recruited 309 university students from universities in Canada and Japan. The Canadian sample consisted of both European-Canadians (Canadians who identified being of European heritage), and Asian-Canadians (Canadians who identified being of East Asian heritage). All of the Japanese sample reported being of Japanese heritage. Because we asked the participants to wear an actigraphy watch for one week (described in the procedures section below), we retained the data for people who provided at least 4 days’ worth of data (i.e. over half of a week). This led us to eliminate 13 participants. The resulting sample size was 295, in which 131 were European-Canadian (*M*_age_ = 22.83, *SD* = 7.64; 36 who identified as men, 92 who identified as women, and 3 who did not respond), 67 were Asian-Canadian *M*_age_ = 19.87, *SD* = 2.03; 10 who identified as men, 54 who identified as women, and 3 who did not respond), and 97 were Japanese students (*M*_age_ = 20.64, *SD* = 2.26; 66 who identified as men, 31 who identified as women. The participants ranged from 17 to 60 years of age (overall *M* = 21.44, *SD* = 5.48).

### Materials

Participants received the AW-Score actigraphy watch from Respironics, which samples at a rate of 32 Hz, and records at one minute epochs. The same set of watches was used in both countries, and sleep duration and efficiency were calculated in identical ways. This data allows one to infer and measure sleep in real-time based on wrist and arm movements. As per recommended guidelines [75, 79], participants were asked to wear the watch for one week on their non-dominant arm, and to only remove it when they were showering. The raw data was inspected for obvious artifacts, such as long periods of zero movement, or abnormally high activity at unexpected times [80]. As another quality check, participants also separately kept a log of when they slept, which allowed the researchers to ascertain that the sleep periods indicated by the watches approximated the timeframe of their self-reported sleep periods. Past research has shown that actigraphy is a generally appropriate and valid measure of sleep time and wakefulness [81–83]. In particular, several studies have demonstrated the validity of actigraphy for assessing total sleep duration and sleep efficiency [79, 84], which are the two key variables that were analyzed in the present study. Participants also completed a battery of assessments asking them various questions about sleep that explored whether people from the two cultural groups had similar attitudes towards ideal sleep durations and the relation between sleep and health. At the end of the study period, the participants indicated their health over the study period. All materials were translated from English into Japanese by translators and then the translations were further examined by one of the authors. Any issues with the translations were resolved by discussions with the original translators.

#### Actigraphy data

We obtained several key variables from the participants’ actiwatches from Respironics. This entailed sleep duration (measured in minutes) and sleep efficiency (i.e., the percentage of time spent in bed sleeping, measured in percentages). Participants also received three daily reminders from their actiwatches (at 10 am, 1 pm, and 9 pm) soliciting their self-reported sleepiness on the watch using a scale from 0 (not at all sleepy) to 4 (extremely sleepy). We did not segregate the data by the day of the week.

#### Nap times

Part way into the study we added a measure of the amount of daily napping for participants to fill out each day of the week. As a result, we only have data for 216 participants.

#### Sleep quality

Participants rated their current quality of sleep on a scale of 1 (Extremely poor) to 7 (Extremely high).

#### Cultural ideal sleep

Participants answered the open-ended question, “According to my primary culture, people should sleep for ____ hours every night.”

#### Personal ideal sleep

Participants answered the open-ended question, “How many hours of sleep would you ideally like to have?”

#### Relationship between sleep and health

Participants indicated, on a scale of -2 to +2, their culture’s perception of the relationship between the amount of sleep and physical health. -2 indicates a strong negative relationship, -1 indicates a weak negative relationship, 0 indicates no relationship, +1 indicates a weak positive relationship, and +2 indicates a strong positive relationship.

#### Health

Participants completed the somatization subscale of the Brief Symptom Inventory [85] which indexes their frequency of experiencing various physical symptoms over the previous week. For this measure, they indicated their frequency of experiencing a variety of symptoms including headaches, shortness of breath, and stomach ache or pain on a 7-point scale (1 = not at all, 7 = very frequently). This measure was completed twice: once at the beginning, and once at the end of the study. Because higher scores indicate poorer health on this measure, this variable was reverse-coded during analysis such that higher score means better health.

### Procedures

Participants first answered a series of questions about various perceptions of sleep. They then wore the actiwatch on their non-dominant hand for seven days. During that time, the watch sent three daily reminders to participants to solicit ratings of sleepiness. After one week, the participants returned the watch and completed the health measure. From the watch, we extracted average sleep duration, and percentage of sleep time sleeping as the primary variables of interest. The dataset and codebook are available at https://osf.io/rn3a4 and at https://osf.io/387fa, respectively.

## Results

All analyses (with the exception of sleepiness) were linear regressions with each criterion variable regressed onto cultural group, which was dummy coded. All regression coefficients are accompanied by percentile bootstrapped 95% confidence intervals (CIs) with 5999 resamples, which leads to better estimations of confidence intervals [86]. The one exception was for sleepiness as measured by participants entering a score into the watch, which was analyzed using hierarchical linear modeling with the various sleepiness reporting periods nested within participants. Table 1 provides a summary of the cultural comparisons for all of the variables, whereas the correlations among the variables are shown in Table 2. Due to the fact that the sample had such a wide distribution for participants’ age, and that older people tend to sleep differently than younger people [87], we included age as a covariate in all analyses. We also note that we have repeated all analyses by excluding the 2 outliers who were aged 55 and 60 and all of the significant effects remain significant. Likewise, because our samples varied in terms of the proportion of women, we also included sex as a covariate in all analyses. The analyses remain largely the same if we do not include the covariates of age and sex.

**Table 1:** Cultural comparisons of all dependent measures.

	European Canadian	Asian Canadian	Japanese
**Sleep Duration**	6 hours, 52.28 minutes^a^	7 hours, 9.41 minutes^a^	6 hours, 5.88 minutes^b^
**Sleep Efficiency**	83.81%^a^	83.49%^a^	81.23%^b^
**Time Napping**	6.03 minutes^a^	17.46 minutes^b^	21.49 minutes^b^
**Sleepiness (0–4 scale)**	2.31^a^	2.05^b^	1.93^b^
**Sleep Quality (1–7 scale)**	4.39^a^	4.07^a^	4.16^a^
**Cultural Ideal Sleep**	7 hours 57.57 minutes^a^	7 hours 41.41 minutes^b^	6 hours 48.60 minutes^c^
**Personal Ideal Sleep**	7 hours, 47.53 minutes^a^	7 hours, 48.09 minutes^a^	7 hours, 26.32 minutes^b^
**Perceived Relation of Sleep and Health (-2 –+2 scale)**	1.65^a^	1.76^a^	0.88^b^
**Health (Time 2 Controlling for Time 1) (1–7 scale, reverse coded)**	5.77^a^	5.89^ab^	5.98^b^

Columns with different superscripts differ at *p* < .05.

**Table 2:** Correlation matrix of all variables.

	Sleep duration	Sleep Efficiency	Time Napping	Sleepiness	Sleep quality	Cultural Ideal Sleep	Personal ideal sleep	Perceived relation of sleep and health	Health (Time 2)
**Sleep duration**	**1.00**								
**Sleep Efficiency**	0.42***	**1.00**							
**Time napping**	-0.14*	-0.05	**1.00**						
**Sleepiness**	0.09	0.10	-0.02	**1.00**					
**Sleep quality**	0.05	0.06	-0.19**	-0.13*	**1.00**				
**Cultural Ideal Sleep**	0.22***	0.17**	-0.23**	0.17**	0.03	**1.00**			
**Personal ideal sleep**	0.25***	0.08	0.03	0.06	0.06	0.36***	**1.00**		
**Perceived relation of sleep and health**	0.12*	0.15*	0.00	0.12 ∙	-0.04	0.29***	0.20**	**1.00**	
**Health (Time 2)**	-0.05	-0.09	0.06	-0.24***	0.26***	-0.06	-0.07	-0.06	**1.00**

* *p* < .05

** *p* < .01

*** *p* < .001.

### Sleep duration

Predicting sleep duration using cultural group, with age and sex as covariates, was significant, *R*^*2*^ = 0.09, *F*(4, 266) = 7.72, *p* < .001. Consistent with past cross-national studies (e.g., [35, 36]), Japanese participants slept significantly less (*M* = 6 hours, 5.88 minutes, *SE* = 9.58 minutes) than the European-Canadian (*M* = 6 hours, 52.28 minutes, *SE* = 11.45 minutes; β = -0.54, *p* < .001, 95% CI [-0.80, -0.28]) and Asian-Canadian participants (*M* = 7 hours, 9.41 minutes, *SE* = 10.76 minutes; β = -0.73, *p* < .001, 95% CI [-1.14, -0.44]). On the other hand, at odds with past cross-national studies, the European-Canadians and Asian-Canadians did not differ significantly (β = 0.19, *p* = .211, 95% CI [-0.16, 0.59]).

### Sleep efficiency

Predicting sleep efficiency (i.e., the percent of time in bed spent sleeping) based on cultural group was significant, *R*^*2*^ = 0.07, *F*(4, 265) = 6.48, *p* < .001. Somewhat surprisingly, the Japanese participants spent significantly *less* amount of their time in bed actually sleeping (*M* = 81.23%, *SE* = 0.63%) than European-Canadian (*M* = 83.81%, *SE* = 0.75%; β = -0.45, *p* < .002, 95% CI [-0.76, -0.13]) and Asian-Canadian participants (*M* = 83.49%, *SE* = 0.95; β = -0.40, *p* < 0.023, 95% CI [-0.76, -0.03]). The latter two did not differ significantly from each other (β = -0.06, *p* = 0.723, 95% CI [-0.33, 0.23]).

### Nap times

Regressing nap times onto cultural group was significant, *R*^*2*^ = 0.06, *F*(4, 195) = 4.45, *p* = .002. The Japanese participants napped significantly more (*M* = 21.49 minutes, *SE* = 3.35 minutes) than European-Canadian participants (*M* = 6.03 minutes, *SE* = 3.41 minutes; β = 0.67, *p* < .001, 95% CI [0.31, 1.03]), but not differently than Asian-Canadian participants (*M* = 17.46 minutes, *SE* = 4.68 minutes; β = 0.17, *p* = .429, 95% CI [-0.41, 0.71]). Asian-Canadian participants napped significantly more than European-Canadian participants (β = 0.49, *p* = .007, 95% CI [0.07, 0.98]).

### Sleepiness

Participants generally report feeling the sleepiest in the morning (*M* = 2.29, *SE* = 0.07) than either in the afternoon (*M* = 1.89, *SE* = 0.07; β = 0.40, *p* < .001, 95% CI [0.29, 0.49]) or in the evening (*M* = 2.04, *SE* = 0.07; β = 0.25, *p* < .001, 95% CI [0.15, 0.35]). The latter two times of day differed significantly from each other as well (β = 0.14, *p* = .006, 95% CI [0.04, 0.25]). Unexpectedly, Japanese participants reported significantly *less* sleepiness (*M* = 1.93, *SE* = 0.08) than European-Canadian (*M* = 2.31, *SE* = 0.09; β = -0.37, *p* < .001, 95% CI [-0.57, -0.17]) but being similarly sleepy to Asian-Canadian (*M* = 2.05, *SE* = 0.12; β = -0.123, *p* = .340, 95% CI [-0.35, 0.13]) participants, regardless of time of day. The latter two groups also differed significantly from each other (β = -0.26, *p* = .019, 95% CI [-0.47, -0.04]). The interactions between cultural groups and times of day did not reach significance (|β|’s < 0.26, *p*’s > .05), and did not affect the generalized effects, indicating that cultural differences did not change significantly as a function of time of day. The one exception is the interaction term comparing European-Canadian and Japanese participants as sleepiness changed from the afternoon to the evening. The contrast showed that the cultural difference was even more pronounced (β_interaction_ = -0.24, *p* = .042, 95% CI [-0.50, -0.00]). In other words, as time went from the afternoon to the evening, sleepiness was even lower for Japanese participants relative to the European-Canadians.

### Sleep quality

Regressing self-rated sleep quality onto cultural group and age was not significant, *R*^*2*^ = 0.00, *F*(4, 283) = 1.24, *p* = .293. Japanese participants had similar sleep quality (*M* = 4.16, *SE* = 0.15) to European-Canadian participants (*M* = 4.39, *SE* = 0.17; β = -0.17, *p* = .245, 95% CI [-0.46, 0.21]), and Asian-Canadian participants (*M* = 4.07, *SD* = 0.122; β = 0.07, *p* = .700, 95% CI [-0.42, 0.29]). The latter two were also not significantly different (β = -0.24, *p* = .129, 95% CI [-0.56, 0.07]).

### Cultural ideal sleep

Predicting cultural ideal sleep using cultural group and age was significant, *R*^*2*^ = 0.36, *F*(4, 283) = 41.79, *p* < .001. Japanese participants’ perceived cultural ideal sleep duration was significantly less (*M* = 6 hours, 48.60 minutes, *SE* = 4.51 minutes) than European-Canadians’ (*M* = 7 hours, 57.57 minutes, *SE* = 5.39 minutes; β = 1.35, *p* < .001, 95% CI [-1.57, -1.12]) and Asian-Canadians’ (*M* = 7 hours, 41.41 minutes, *SE* = 6.92 minutes; β = 1.03, *p* < .001, 95% CI [-1.33, -0.69]). The latter two were significantly different from each other (β = -0.32, *p* = .013, 95% CI [-0.62, -0.09]).

### Personal ideal sleep

Predicting personal ideal sleep using cultural group and age was significant, *R*^*2*^ = 0.11, *F*(4, 272) = 9.54, *p* < .001. Japanese participants idealized less sleep (*M* = 7 hours, 26.32 minutes, *SE* = 5.95 minutes) than European-Canadian (*M* = 7 hours, 47.53 minutes, *SE* = 7.05 minutes; β = -0.38, *p* < .007, 95% CI [-0.66, -0.09]) and Asian-Canadian participants (*M* = 7 hours, 48.09 minutes, *SE* = 9.19 minutes; β = -0.39, *p* < .022, 95% CI [-0.73, -0.04]). The latter two did not differ significantly from each other (β = 0.01, *p* = .948, 95% CI [-0.26, 0.30]).

### Sleep and health

Predicting the perceived relationship between sleep and health using cultural group and age was significant, *R*^*2*^ = 0.15, *F*(4, 283) = 13.48, *p* < .001. Japanese participants perceived a significantly weaker link between sleep and health (*M* = 0.88, *SE* = 0.09) than did European-Canadian (*M* = 1.65, *SE* = 0.11; β = -0.87, *p* < .001, 95% CI [-1.12, -0.59]) and Asian-Canadian participants (*M* = 1.76, *SE* = 0.14; β = -0.99, *p* < .001, 95% CI [-1.27, -0.72]). The latter two did not differ from each other (β = 0.13, *p* = .393, 95% CI [-0.12, 0.38]).

### Health

We report the analyses with self-reported health in 3 ways; subjective health (in terms of the absence of physical symptomology) regressed on to cultural group and age, and gender at Time 1 (time of first arrival in the lab), at Time 2 (when participants returned to the lab at the end of the study), and then at Time 2 controlling for Time 1. Regressing subjective health on cultural group was significant at Time 1, *R*^*2*^ = 0.02, *F*(4, 277) = 2.72, *p* = .030. The Japanese participants (*M* = 5.70, *SE* = 0.11) did not differ from the European-Canadian participants (*M* = 5.70, *SE* = 0.11; β = -0.01, *p* = .921, 95% CI [-0.28, 0.25]), or the Asian-Canadian participants (*M* = 5.72, *SE* = 0.14; β = 0.02, *p* = .924, 95% CI [-0.34, 0.35]). The latter two did not differ significantly from each other either (β = 0.03, *p* = .842, 95% CI [-0.30, 0.35]). The significance in this model appeared to be driven primarily by age, such that older participants curiously reported better health (β = 0.16, *p* < .007, 95% CI [0.08, 0.28]).

Regressing subjective health onto cultural group and age was also significant at Time 2, *R*^*2*^ = 0.02, *F*(4, 276) = 2.69, *p* = .031. The Japanese participants had marginally better self-reported health (*M* = 6.04, *SE* = 0.09) than European-Canadian participants (*M* = 5.84, *SE* = 0.11; β = 0.25, *p* = .095, 95% CI [-0.04, 0.53]), but not from Asian-Canadian participants (*M* = 5.97, *SE* = 0.14; β = 0.08, *p* = .641, 95% CI [-0.26, 0.43]). The latter two did not differ significantly from each other (β = -0.17, *p* = .292, 95% CI [-0.49, 0.16]). Similar to the previous model, health at Time 2 was predicted significantly by age in the same direction (β = 0.16, *p* = .007, 95% CI [0.02, 0.24]).

Regressing subjective health at Time 2 onto cultural group, age, and health at Time 1 was also significant, *R*^2^ = 0.44, *F*(5, 270) = 43.69, *p* < .001. Unsurprisingly, health at Time 1 significantly predicts health at Time 2 in a positive manner, (β = 0.66, *p* < .001, 95% CI [0.54, 0.77]). The Japanese participants had significantly better self-reported health (*M* = 5.98, *SE* = 0.07) than European-Canadian participants (*M* = 5.77, *SE* = 0.08; β = 0.26, *p* = .026, 95% CI [0.05, 0.46]), but not from Asian-Canadian participants (*M* = 5.89, *SE* = 0.11; β = 0.12, *p* = .389, 95% CI [-0.10, 0.33]). The latter two did not differ significantly from each other (β = -0.14, *p* = .252, 95% CI [-0.09, 0.37]). That is, after controlling for subjective health at Time 1, at the end of the study Japanese reported better health than European-Canadians, with Asian-Canadians reporting subjective health that was intermediate to the other two.

## General discussion

The present study points to some curious findings. First, we found that Japanese university students sleep less than Canadian university students. This rather pronounced cultural difference in sleep duration is consistent with some other cross-cultural comparisons (e.g., [35–37]), and is not easy to dismiss in terms of other accounts. For instance, while many have noted the Japanese penchant for napping (e.g., [66]), this cannot account for the cultural differences that we observed in their night time sleep duration. Although, on average, our Japanese sample napped for 15 minutes and 4 minutes longer than our European-Canadian and Asian-Canadian samples, respectively, the magnitude of these amounts are dwarfed by the differences in nighttime sleep where our Japanese sample slept 46 minutes and 64 minutes shorter than our European-Canadian and Asian-Canadian samples, respectively. Second, this difference in sleep duration cannot be accounted for by the speculative notion that Japanese are somehow sleeping more efficiently than Canadians. Rather, the opposite was true; our Japanese sample spent a *lower* percentage of their time in bed actually sleeping than either of our Canadian samples, thereby amplifying the magnitude of the cultural difference in the total amount that people slept. Third, while some have raised the question of whether biological differences between ethnicities may be relevant for interpreting cross-national differences in sleep duration (e.g., [37]), our findings would seem to speak against this. While East Asian samples are consistently found to be at the low end of sleep duration in cross-cultural studies (e.g., [36]), our Asian-Canadian sample looked remarkably similar to our European-Canadian sample in terms of their sleep behaviors and beliefs–this suggests that people acculturate to local cultural norms in terms of sleep duration. Fourth, it does not appear to be that Japanese are sleeping less than Canadians due to the results of additional obligations that keep them up at night; when asked how much they would ideally like to sleep, Japanese prefer a shorter sleep duration than Canadians. This latter finding is consistent with a claim that common attitudes towards sleep in a culture are related to people’s sleep behaviors [13, 61].

But despite this pronounced cultural difference in sleep duration, our Japanese sample curiously did not appear to be suffering from their reduced sleep compared with the Canadians; in particular, the Japanese reported being *less* sleepy than European-Canadians. It is possible that this cultural difference in self-reported sleepiness is due to Japanese possessing more of a stoic self-presentational norm by not reporting any negative experiences. However, this alternative explanation is undermined by accounts that expressing fatigue and public napping are commonplace and accepted in Japanese society [66]. Moreover, the subjective health data did not find that Japanese had any worse self-reported health than Canadians, and in fact had better subjective health at the end of the study (controlling for health at the beginning of the study) than did the European-Canadians. It is intriguing that our Japanese participants not only showed fewer negative consequences to their average shorter sleep durations compared with Canadians, but they also reported believing that sleep duration was less closely tied to health outcomes than Canadians. Unless there are cultural differences in the health of our samples that were not captured with our self-report measure, it appears that overall our Japanese sample did not seem to be suffering from the expected consequences of shorter average sleep durations; they appeared to somehow need less sleep than our Canadian samples.

The finding that Japanese sleep less than people from other countries, yet do not seem to be suffering from the harmful outcomes that have been linked with reduced sleep duration that are captured with our self-report measures, is puzzling. The highly similar sleep durations and questionnaire responses between the Asian-Canadian and European-Canadian samples makes it unlikely that the observed cross-national differences are due to any innate differences between the different ethnicities (cf., [37]). Sleep clearly serves important biological functions, and the observed cross-national differences raise the possibility that habitual cultural practices may shape aspects of human biology, which is a perspective that has been increasingly noted in the literature [88–91]. It’s possible that the relatively better health outcomes of Japanese can be accounted for by the existence of other cultural protective factors (such as dietary practices; e.g., [60]) which may conceal the ways that Japanese samples are suffering from their abbreviated sleep. Another speculative possibility is that the harmful consequences of shorter sleep duration may derive, in part, from sleeping less than widely shared cultural norms for sleep. This speculation is in keeping with the findings that living at odds with local cultural norms has been found to be associated with a variety of other negative health consequences, such as subjective well-being [92], healthy eating [93], and better immune response [94].

### Limitations

There are a number of different variables that may have influenced participants’ sleep duration which we did not control for in this study. Some of these relate to the sleep environment that participants were in, such as the ambient temperature in participants’ bedrooms, bedding, bed-sharing, light pollution, and geographic location. Other potential confounding variables include those related to participants’ lifestyles, such as electronic screen time, amount of daily activity, work schedules, diet, body mass index, and preexisting health conditions. Since we did not assess these environmental and lifestyle variables, we are not able to account for the impact that they had on our participants’ sleep behaviors, and it is possible that some of these confounding variables contributed to our observed findings.

Our samples are limited to university students in Canada and Japan. It remains unclear whether we would have obtained similar patterns had we targeted other samples, however, the fact that parallel kinds of cultural differences in sleep duration have been observed in a wide variety of different age groups suggests that the findings might not be limited to university students.

Our self-report measures of physical health are surely not comprehensive and their subjective nature reduces their validity. Future research would benefit by incorporating more extensive measures of physical health, or use biomarkers of health. Moreover, understanding whether sleep architecture varies across cultures as a function of local sleep norms would be informative. Likewise, our cross-cultural comparisons of sleep duration may conceivably be influenced by potential response style biases (e.g., [95, 96]), with the resulting limitations of interpreting differences in self-reported means.

While the cultural differences obtained in the present data are intriguing, the various limitations of our findings prevent us from drawing firm conclusions with respect to why these cultural differences in sleep duration exist. Future research that compares sleep duration and health across many countries while controlling for various other differences in nutrition and lifestyle may shed light on the degree to which national differences in sleep duration are related to health outcomes.
